# Effect of Extra Virgin Olive Oil High in Bioactive Compounds on Atherosclerosis in Apoe‐Deficient Mice

**DOI:** 10.1002/mnfr.70223

**Published:** 2025-09-01

**Authors:** Roberto Martínez‐Beamonte, Cristina Barranquero, Laura Soler, Tania Herrero‐Continente, Anny Carolina Rondón, Carmen Arnal, Gloria Estopañán, Roberto Lasheras, María Jesús Rodriguez‐Yoldi, Joaquín Carlos Surra, Olga Martín‐Belloso, Isabel Odriozola‐Serrano, Jesús Osada, María Ángeles Navarro

**Affiliations:** ^1^ Departamento de Bioquímica y Biología Molecular y Celular Facultad de Veterinaria Instituto de Investigación Sanitaria de Aragón Universidad de Zaragoza Zaragoza Spain; ^2^ Instituto Agroalimentario de Aragón CITA‐Universidad de Zaragoza Zaragoza Spain; ^3^ CIBER de Fisiopatología de la Obesidad y Nutrición Instituto de Salud Carlos III Madrid Spain; ^4^ Departamento de Patología Animal Facultad de Veterinaria Universidad de Zaragoza Zaragoza Spain; ^5^ Centro de Investigación y Tecnología Agroalimentaria de Aragón (CITA), Avda Zaragoza Spain; ^6^ Laboratorio Agroambiental Servicio de Seguridad Agroalimentaria de la Dirección General de Alimentación y Fomento Agroalimentario, Gobierno de Aragón Zaragoza Spain; ^7^ Departamento de Farmacología, Fisiología y Medicina Legal y Forense, Facultad de Veterinaria, Instituto de Investigación Sanitaria de Aragón Universidad de Zaragoza Zaragoza Spain; ^8^ Departamento de Producción Animal y Ciencia de Los Alimentos, Escuela Politécnica Superior de Huesca, Instituto de Investigación Sanitaria de Aragón Universidad de Zaragoza Zaragoza Spain; ^9^ Department of Food Technology, Engineering and Science University of Lleida Lleida Spain; ^10^ Agrotecnio‐CERCA Center Lleida Spain

**Keywords:** apolipoprotein E‐deficient mice, atherosclerosis, EVOO, lipoproteins, squalene

## Abstract

To test the effects of extra virgin olive oil (EVOO) enriched in specific bioactive compounds (EVOO HBC) on atherosclerosis and fatty liver, three isocaloric Western diets differing in the type of fat (palm, EVOO, or EVOO HBC) were fed to *Apoe*‐deficient mice for 12 weeks. Plasma lipids, lipoprotein characterization, circulating CD36‐expressing monocytes, and M2 peritoneal macrophages were quantified. Hepatic squalene and cross‐sectional and *en face* atherosclerotic lesions were analyzed. Compared to the palm group, plasma triglyceride and glucose levels increased, while APOA1, paraoxonase 1 activity, and lipoprotein oxidation decreased in mice fed both EVOO groups. The latter stored liver squalene according to the amount consumed. *En face* and cross‐sectional atherosclerotic lesions were lower in the EVOO groups. CD36 expression in circulating monocytes was lower and M2 peritoneal macrophages were higher in the EVOO groups. In males, there was a reduced presence of CD68‐expressing cells in atherosclerotic plaques, while in females, there was a reduction in *en face* lesions that negatively correlated with high‐density lipoprotein (HDL)‐phospholipid efflux. The recruitment of macrophages into atherosclerotic plaques and the improvement of HDL efflux may be sex‐dependent and attributable to the high content of squalene and a specific oleuropein aglycone.

## Graphical abstract

EVOO with high content of squalene and the dialdehyde form of decarboxymethyl oleuropein aglycone in HBC consumed by mice reduced atherosclerotic foci and growth of plaques in both sexes. These findings were accompanied by decreases in APOA1, paraoxonase 1, lipoprotein oxidation, expression of CD36 in monocytes, increased differentiation of macrophages into the M2 phenotype, and hepatic squalene. This new EVOO modulates the recruitment of macrophages or improves HDL efflux in a sex‐dependent way.

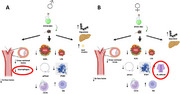

## 1 Introduction

The Mediterranean diet, from its earlier epidemiological studies, was found to be associated with low mortality from cardiovascular diseases [1, 2, 3]. In this dietary pattern, the main source of fat, with more than 25% of the total caloric intake, was virgin olive oil (VOO) [2, 4]. In fact, recent interventions comparing Mediterranean diets containing VOO versus low‐fat diets have evidenced low cardiovascular mortality of the former diets either in primary [5] or secondary [6] prevention. To explain these findings, a wide range of studies have shown that virgin olive oil consumption modulates in a positive way endothelial dysfunction, blood pressure, insulin resistance, thrombosis, and lipid metabolism [4, 7].

The highest quality preparation of VOO is known as extra‐virgin olive oil (EVOO), and both are composed of triglycerides containing mainly oleic acid and a minor fraction representing 0.5%–1.5% of oil named unsaponifiable [8]. This constitutes a chemically diverse mixture where hydrocarbons, triterpenes, phytosterols, and phenolic compounds are present. These biologically active compounds are considered to be responsible for the VOO properties [4, 8, 9, 10]. Given this preeminent role of these compounds, the development of novel extraction systems and the enrichment of EVOO with additional bioactive compounds represent a promising research area that has the potential to contribute to the formulation of functional foods and nutraceuticals [11]. In this regard, Klikarová et al. estimated that only 2% of the olive phenols are transferred to the EVOO, which is composed of more than 36 different structurally distinct phenolic compounds [12]. Accordingly, the EVOO extraction process is constantly evolving, applying new techniques and processes that increase efficiency, reduce extraction time, and slightly modify the EVOO composition. In this regard, high‐speed centrifugation of olive paste resulted in an oil enriched in minor components showing a great anti‐atherosclerotic effect [13]. High power ultrasound treatment of olive paste increased its green sensorial attribute [14]. High vacuum conditions during the malaxation of Picual VOO resulted in an increased oleacein content [15]. The use of electric pulse increased the amount of phytosterols in EVOO [16]. These examples illustrate that the existing extraction systems for obtaining EVOO could be enhanced to produce EVOO of higher content in bioactive compounds and consequently more functional. In line with this, the present study was designed to compare two EVOOs from the Koroneiki variety, one prepared following the standard procedure (EVOO) and the other prepared using a new patented extraction procedure designed to maximize the recovery of bioactive compounds and stability, named EVOO‐HBC [17]. Both EVOO were provided as purified Western diets to Apoe‐deficient mice to compare their effect on the atherosclerotic development of this model.

## 2 Material and Methods

### 2.1 Animals and Experimental Procedure

*Apoe*‐deficient mice on the C57BL/6J genetic background were obtained from Charles River (Charles River Laboratories, Barcelona, Spain) and subsequently bred at the Centro de Investigación Biomédica de Aragón (CIBA) in Zaragoza, Spain. In order to establish groups with similar initial body weight and plasma cholesterol levels, 9‐ to 10‐week‐old mice (60 males and 60 females) were weighed, blood samples taken from the facial vein (following an overnight fast), and their cholesterol levels were analyzed. Six groups of *Apoe*‐deficient mice were established, three groups of males and another three of females, and housed in sterile filter‐top cages in rooms maintained on a 12‐hour light/12‐hour dark cycle in the CIBA. All mice had ad libitum access to food and water. Feed consumption was monitored on a weekly basis within each cage to calculate the individual daily feed intake of each mouse. In addition, body weight was measured every 2 weeks throughout the 12‐week dietary intervention period. The mouse experiments were conducted in accordance with the EU Directive 2010/63 on the protection of animals used for scientific purposes, and the study protocol was approved by the Animal Ethics Committee of the University of Zaragoza (PI61/18).

Four males and five females of each group were sacrificed at 11 weeks of diet intervention, in order to analyze the blood monocytes and peritoneal macrophages. At the conclusion of the experiment in Week 12, feed was withdrawn for 16 h, and the mice were weighed and euthanized by suffocation in a CO_2_ chamber. Blood samples were collected by cardiac puncture, and plasma and serum were separated by centrifugation at 3000 × *g* for 10 min. The livers were rapidly obtained, frozen in liquid nitrogen, and stored at −80°C until processing. An aliquot was preserved in buffered formaldehyde for histological analysis. The hearts and aortas were then perfused with phosphate‐buffered saline (PBS), after which the hearts were filled with OCT Tissue‐Tek (Sakura Finetek, Barcelona, Spain). These were then frozen in liquid nitrogen and stored at −80°C, and dissected aortas were stored in buffered 10% formaldehyde at 4°C.

### 2.2 Diets

During the intervention, the mice were fed three purified Western diets containing 20% fat and 0.15% cholesterol, which differed in the source of fat as follows: refined palm oil provided by Gustav Heess (Santa Perpètua de Mogoda, Spain), EVOO standard (EVOO), or EVOO high in bioactive compounds (EVOO HBC) provided by Cleanthi Alpha Olenic LTD (Larnaca, Cyprus). The preparation of the latter EVOO followed the patented procedure PCT/GB2015/053714 [17]. This patent encompasses numerous processes intended to ensure optimal conditions for the olives, including refrigeration and washing immediately prior to milling without compromising the integrity of the pits and their contents. The pulp is then malaxed in a bioreactor without the addition of water or exposure to atmospheric oxygen in a process known as “zero water, zero oxygen.” This inhibits the oxidation of oil phenols by polyphenol oxidase during malaxing, which takes approximately 60 min. The oil is then separated by centrifugation and filtered within 48 h, eliminating all traces of water emulsions, olive pulp, and enzymes. This process is intended to maximize the stability and bioactive compounds of the EVOO.

These three diets were formulated in our laboratory in accordance with the recommendations of the Nutrient Requirements of Laboratory Animals [18] and their components have been previously described [19]. Post‐preparation, the diets were immediately frozen, lyophilized, and stored at −20°C in vacuum‐sealed bags until use.

### 2.3 Analysis of Dietary Fatty Acids, Tocopherols, and Phenolic Compounds

The fatty acid profile was determined by gas chromatography in accordance with the official method approved by the European Commission [20]. The identification and quantification of the α‐, γ‐, and δ‐tocopherol isomers were performed by reverse‐phase high‐performance liquid chromatography with a fluorescence detector (Kontron Instruments, Eching, Germany) [21]. The phenolic extract of VOO was obtained according to the procedure described by Montedoro et al. [22]. The quantification and identification of the individual phenolic compounds were carried out by UPLC‐MS/MS on an AcQuity Ultra‐PerformanceTM liquid chromatography with tandem mass spectrometry system (Waters, Milford, Massachusetts, USA). The chromatographic conditions were those described by Delpino‐Rius et al. [23]. The quantification of the individual phenolic compounds was calculated using calibration curves of commercial standards.

### 2.4 Plasma Parameters

Total plasma cholesterol, glucose, and triglyceride concentrations were measured using a microtiter assay with a commercial Infinity kit (Thermo Scientific, Madrid, Spain) and a glucose kit (BioSystems, Barcelona, Spain), following the manufacturers’ instructions. Total serum apolipoprotein A1 (apoA‐1) was quantified via ELISA, and paraoxonase 1 (PON1) activity was measured as previously described by Navarro et al. [24] and by Martinez‐Beamonte et al. [25]. The plasma lipoprotein profile was determined in 100 µL of pooled plasma samples from each group and sex by fast protein liquid chromatography (FPLC) gel filtration using a Superose 6B column (GE Healthcare, Chicago, Illinois, USA) as previously described [25]. The plasma determination of the HDL‐specific phospholipid efflux (HDL‐SPE) and the non‐specific cholesterol efflux capacity (NS‐CEC) was determined as described by Sato et al. [26].

### 2.5 Evaluation of Atherosclerotic Lesions

*En face* analyses of dissected aortas and the cross‐sectional analyses of aortic roots and aortic lesion characteristics were carried out as previously described [19].

### 2.6 Hepatic Histological Analyses

Liver specimens stored in buffered formaldehyde were embedded in paraffin, and sections (4 µm) were stained with hematoxylin and eosin. A Zeiss Axioscan.Z1 (Zeiss, Oberkochen, Germany) slide scanner was utilized to image all specimens. The evaluation of lipid droplets was conducted by quantifying their areas in each liver section utilizing Adobe Photoshop CS3 (Adobe Inc. San Jose, California, USA). These areas were expressed as a percentage of the total liver section, as previously described by Guillen et al. [27].

### 2.7 Liver Squalene Content

As previously published, the squalene content in each mouse's liver was processed and measured [28].

### 2.8 Analysis of Circulating Blood Monocytes

In the case of peripheral blood, samples were extracted from the facial vein and collected with 5 µL of EDTA. A total of 27 animals (4 male and 5 female mice per group) were utilized in the study. Three pools of 50 µL per experimental group were prepared, along with separate tubes for unstained and single‐color controls, which contained the blood of the three additional females. The blood samples were then incubated with 2.5 µL of the corresponding mouse monoclonal conjugated antibodies (CD11B‐VB421 clone M1/70 #101235, CD115‐APC clone AFS98 #135509, and CD36‐A488 clone HM36 #102608 from BioLegend, San Diego, California, USA) for 30 min at 4°C, protected from light. Subsequently, the red blood cells were lysed using 1 mL of Cytognos QuicklysisTm (Cyt‐QL‐1, Cytognos S.L., Salamanca, Spain) for 30 min in a dark environment at room temperature. Following this, the remaining cells were pelleted and resuspended in 300 µL of cold phosphate‐buffered saline (PBS). The samples were then analyzed using a Gallios Flow Cytometer (Beckman Coulter, Brea, California, USA) and the Kaluza analysis software. The initial population was gated based on size and complexity (FSC and SSC). Monocytes were selected as cells showing double positivity for the CD11b and CD115 markers [29]. Statistical analyses were then carried out using the fluorescence intensity of cells CD36+ within the specific gate of monocytes.

### 2.9 Analysis of Peritoneal Macrophages

In order to study peritoneal macrophages, the same animals and groups of the previous section were utilized, with two pools of two animals per group. The peritoneal fluids of the additional female mice of each diet were pooled and utilized as unstained and single‐stained controls, respectively [30]. Immediately following euthanasia of the animals, 4 mL of ice‐cold sterile PBS were injected into the peritoneal cavity, followed by a gentle massage of the abdominal walls. The fluid was then collected, and the cellular components isolated by centrifugation (1500 rpm, 10 min at 4°C). The cells were then incubated in 50 µL of FcR Blocking Reagent (anti‐mouse CD16/CD32) (Miltenyi Biotec, Bergisch Gladbach, North Rhine‐Westphalia, Germany) diluted 1/10 in 1% BSA for 10 min on ice. The collected cells were then stained with the following mouse monoclonal antibodies from BioLegend: CD11B‐VB421 clone M1/70 #101235, F4/80‐APC clone BM8 #123116, CD206‐PE clone C068C2 #141705, and CD36‐A488 clone HM36 #102608. A volume of 2.5 µL of each conjugated antibody was added to each sample (final dilution 1/20) and incubated in the same conditions as the blood samples. The cells were then washed with cold PBS, followed by centrifugation and resuspension in 200 µL of cold PBS. A Gallios Flow Cytometer was then employed, acquiring a minimum of 200 000 events per sample. Each run included unstained and single‐color samples for compensation. Flow cytometry analyses were performed with Kaluza analysis software following the next gating strategy. Initially, a population was excluded on the basis of cell death, based on size and intracellular composition (FSC vs. SSC). Then, double‐positive cells (CD11b high/F4/80 high) were selected as mature peritoneal macrophages. The final gate was used to extract the percentage of CD206+ (M2 marker) cells, and the subpopulation of M2 macrophages were measured.

### 2.10 Statistical Analyses

The data are presented as the mean ± standard deviation. The statistical analysis was performed by ordinary one‐way ANOVA with Tukey multiple comparison test, except for the analysis of the macrophage and monocyte data. For this particular analysis, Mann–Whitney *U* test was utilized because the pooled data had a low *n* value. Statistical analyses were performed with GraphPad Prism 8.0.1 software for Windows (GraphPad, San Diego, California, USA). Statistical significance was considered as *p* < 0.05. Spearman's bivariate correlations were performed using the Statistical Package for Social Sciences version 27 (IBM, Armonk, New York, USA).

## 3 Results

### 3.1 Fatty Acids and Phenolic Compound Profiles of EVOO and EVOO HBC

As shown in Table 1, the EVOOs used have a high oleic acid content, ranging from 73.49% to 75.91% for EVOO and EVOO HBC, respectively. This is in agreement with the fatty acid composition of virgin olive oil as previously reported [8].

**TABLE 1:** Composition of EVOO and EVOO HBC.

Fatty acids (%)	EVOO	EVOO HBC
Miristic (C14:0)	0.01	0.01
Palmitic (C16:0)	13.98	12.77
Palmitoleic (C16:1)	0.94	0.91
Margaric (C17:0)	0.04	0.05
Margaroleic (C17:1)	0.07	0.08
Estearic (C18:0)	3.17	2.9
Oleic (C18:1)	73.49	75.91
Linoleic (C18:2)	5.75	5.06
Linolenic (C18:3)	0.86	0.85
Araquidic (C20:0)	0.54	0.55
Gadoleic (C20:1)	0.34	0.31
Behenic (C22:0)	0.19	0.16
**Minor components**		
Squalene	5081	13 620
		
alfa Tocopherol	320	301
delta Tocopherol	< 0.1	0.3
gamma Tocopherol	8.9	8.8
		
Oleocanthal & Oleacein	850	710
Lignans	54.1	< 3
Oleuropein aglycone (3,4‐DHPEA‐EA)	13.9	< 3
Ligstroside aglycone (p, HPEA‐EA)	9.3	< 3
Dialdehyde form of decarboxymethyl oleuropein aglycone (3,4‐DHPEH‐EDA)	< 3	94.7
Hydroxytyrosol (3,4 DHPEA)	194	67.3
Tyrosol (p, HPEA)	128	28.4
Cyanidin chloride	50.54	< 1
p‐Coumaric acid	143.4	113.1
Ferulic acid	110.2	< 1
Vanillic acid	987.4	234.2

*Note*: Fatty acids are expressed as percentages. Minor components are the mean of triplicate determinations for each compound expressed as mg/kg.

There were some notable differences between the two EVOO samples in terms of minor bioactive compounds (Table 1), such as squalene content, which was found at 5081 and 13 620 mg/kg for EVOO and EVOO HBC, and the dialdehyde form of decarboxymethyl oleuropein aglycone (3,4‐DHPEH‐EDA) was lower than 3 and 94.7 mg/kg for EVOO and EVOO HBC, respectively. All other phenolic compounds, such as oleocanthal and oleacein, lignans, oleuropein aglycone (3,4 DHPEA‐EA), ligstroside aglycone (p, HPEA‐EA), hydroxytyrosol, tyrosol, cyanidin chloride, ferulic acid, and vanillic acid were found at lower levels in EVOO HBC.

### 3.2 Feed Consumption, Body Weight Gain, and Liver Weight

As shown in Figure S1, the feed consumption (Figure S1, panels A and B) revealed that males consumed 2.4 ± 0.2 g/day and females 2.0 ± 0.1 g/day without statistical differences among groups within each sex. These results rule out intake bias among dietary fats. No significant changes were observed for body weight gain (Figure S1, panel C) in both sexes. A clear statistical significance was observed between the EVOO versus EVOO HBC group for liver weight in male mice (Figure S1, panel D).

### 3.3 Liver Parameters

**FIGURE 1:**
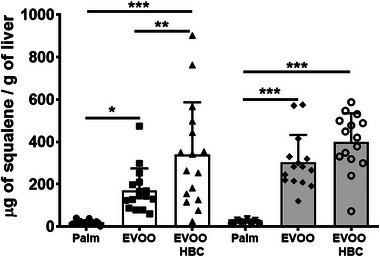
Liver squalene content in the different experimental groups. The bar chart shows the mean and standard deviation for all experimental groups along the individual data. The white boxes correspond to male mice receiving palm oil (*n* = 16), extra virgin olive oil (EVOO, *n* = 16), and high biological compound extra virgin olive oil (EVOO HBC, *n* = 16), while the grey boxes correspond to females (*n* = 15 for all groups). Statistical analysis was performed by ordinary one‐way ANOVA with Tukey multiple comparisons. The significance levels are indicated by * for *p* ≤ 0.05, ** for *p* ≤ 0.01, and *** for *p* ≤ 0.001.

Liver squalene content was determined to verify the bioavailability of the compound in the different experimental groups. The average values recorded were 18, 171, and 338 µg/g for males and 22, 302, and 400 µg/g for females on the palm, EVOO, and EVOO HBC diets, respectively (Figure 1).

**FIGURE 2:**
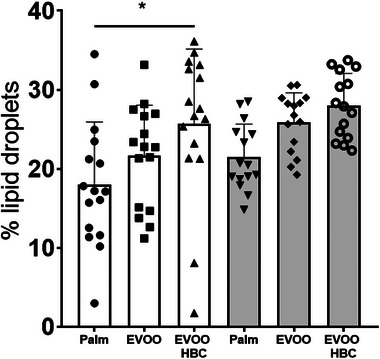
Hepatic fat content according to different diets at the end of the experiment. The results are expressed as a percentage of total area covered by lipid droplets. The bar chart shows the mean and standard deviation for all experimental groups along the individual data. The white boxes in the graph correspond to male mice receiving palm oil (*n* = 16), extra virgin olive oil (EVOO, *n* = 16), and high biological compound extra virgin olive oil (EVOO HBC, *n* = 16), while the grey boxes correspond to females (*n* = 15 for all groups). Statistical analysis was performed by ordinary one‐way ANOVA with Tukey multiple comparisons. The significance levels are indicated by * for *p* ≤ 0.05.

The degree of hepatic steatosis was assessed by measuring the percentage of liver area covered by lipid droplets (Figure 2). In males, a statistically significant increase was observed between those who consumed a palm oil diet and those who consumed EVOO HBC, with no statistical significance in females.

### 3.4 Plasma Parameters

**FIGURE 3:**
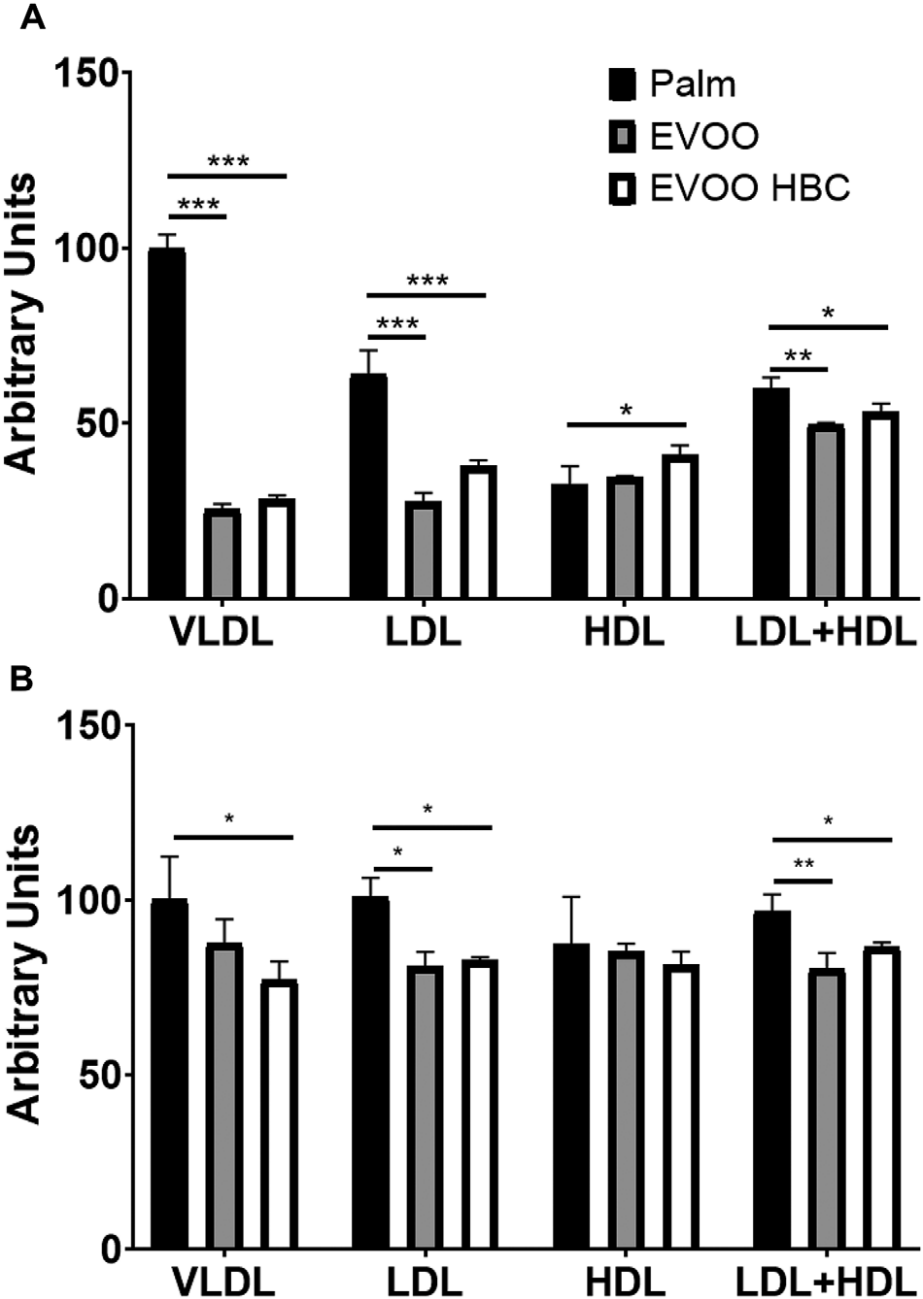
The effects of diverse dietary regimens on the ROS levels of plasma FPLC‐isolated lipoproteins. The bar chart shows the mean and standard deviation for all experimental groups of triplicate determinations of pooled FPLC fractions expressed as arbitrary units of fluorescence. Figure A corresponds to males and Figure B to females. Statistical analysis was performed by ordinary one‐way ANOVA with Tukey multiple comparisons. The significance levels are indicated by *, *p* ≤ 0.05 and **, *p* ≤ 0.01.

The different diets resulted in changes in the plasma parameters shown in Table 2. In both males and females, statistically significant elevated levels of triglycerides and glucose were observed in both EVOO groups. No changes in total cholesterol were observed by the different diets. In agreement with the absence of change in the latter parameter, the lipoprotein profile (Figure S2, panels A and B) exhibited a modest elevation in VLDL total cholesterol in the palm group and a slight increase in LDL in the EVOO groups. As shown in Figure 3, ROS content for both VLDL and LDL revealed a significant increase in males in the palm group (Figure 3A). A similar effect was observed in females, albeit with smaller differences (Figure 3B).

**TABLE 2:** Effect of different diets on plasma parameters.

	Palm	EVOO	EVOO HBC
**Males**			
Triglycerides (mg/dL)	196 ± 33	324 ± 91^**^	373 ± 140^**^
Glucose (mg/dL)	273 ± 43	374 ± 81^**^	369 ± 90^**^
Total cholesterol (mg/dL)	381 ± 64	397 ± 61	389 ± 61
**Females**			
Triglycerides (mg/dL)	284 ± 60	570 ± 130^**^	552 ± 134^**^
Glucose (mg/dL)	198 ± 29	288 ± 77^*^	266 ± 86^*^
Total cholesterol (mg/dL)	362 ± 38	347 ± 49	353 ± 54

*Note*: Data are mean ± SD for each group with *n* = 16 in males and *n* = 15 in females. Unless specified, statistical analysis was carried out using one‐way ANOVA with Tukey multiple comparisons.

^*^ *p* < 0.05.

^**^ *p* < 0.001 versus Western.

**FIGURE 4:**
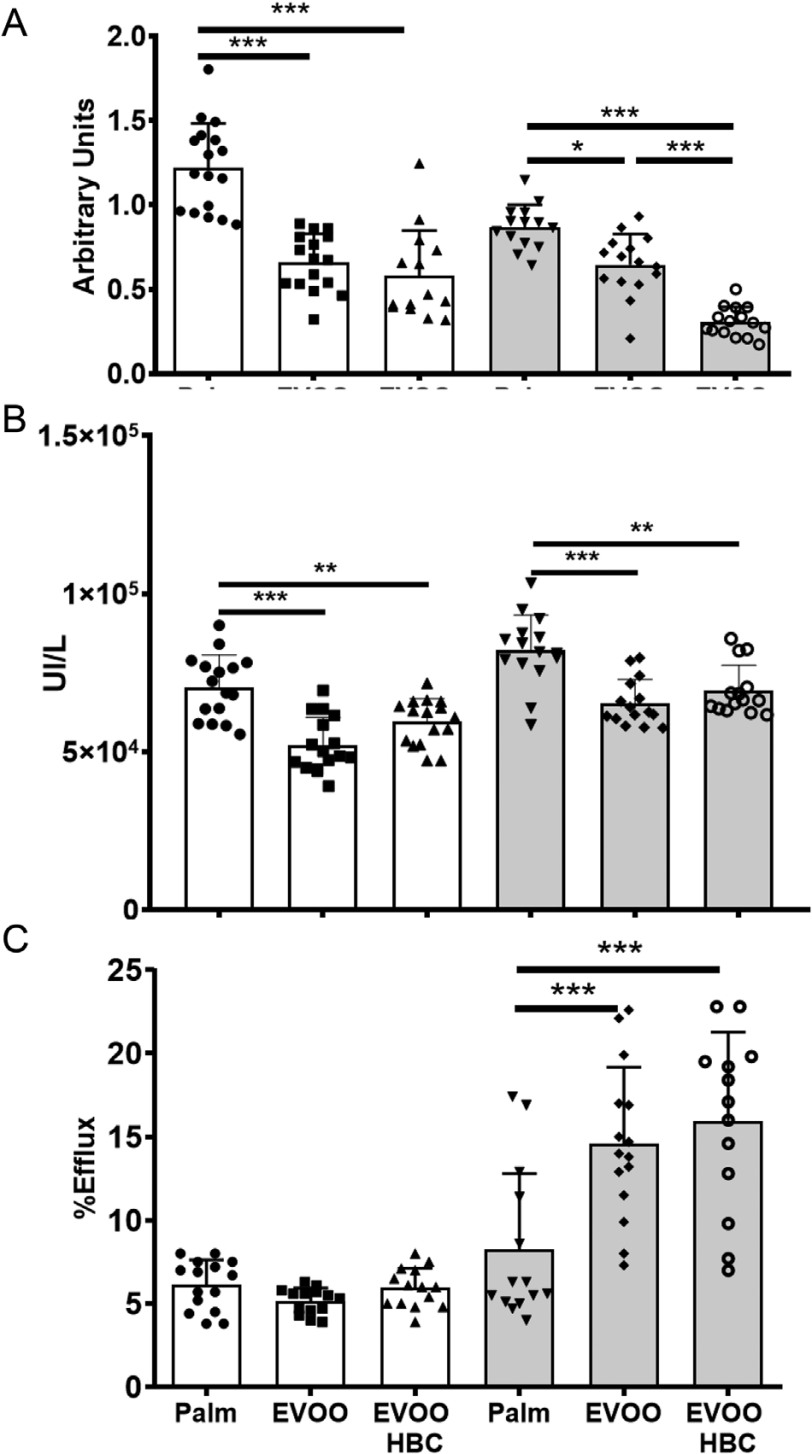
Effects of the different diets on several plasma parameters. (A) APOA1 expressed as arbitrary units, (B) PON1 activity as international units per liter, and (C) HDL‐specific phospholipid efflux expressed as percentage of maximum value. The bar chart shows the mean and standard deviation for all experimental groups along the individual data. The white boxes in the graph correspond to male mice receiving palm oil (*n* = 16), extra virgin olive oil (EVOO, *n* = 16), and high biological compound extra virgin olive oil (EVOO HBC, *n* = 16), while the grey boxes correspond to females (*n* = 15 for all groups). Statistical analysis was performed by ordinary one‐way ANOVA with Tukey multiple comparisons. The significance levels are indicated by * *p* ≤ 0.05, ** *p* ≤ 0.01, and *** *p* ≤ 0.001.

The APOA1, paraoxonase activity (PON1), and potential HDL‐specific phospholipid efflux (HDL‐SPE) are depicted in Figure 4A–C, respectively. Statistically significant increases in APOA1 levels and serum PON1 activity were observed in males and females in the palm group. The HDL phospholipid‐efflux capacities were analyzed using fluorescent particles as described by Sato et al. [26]. In this case, there was a significant increase in females in both EVOO groups. Of particular interest were the elevated levels observed, with values of 8.2%, 14.6%, and 16% for palm, EVOO, and EVOO HBC, respectively, as illustrated in Figure 4C.

### 3.5 Monocyte and Macrophage Analyses

**FIGURE 5:**
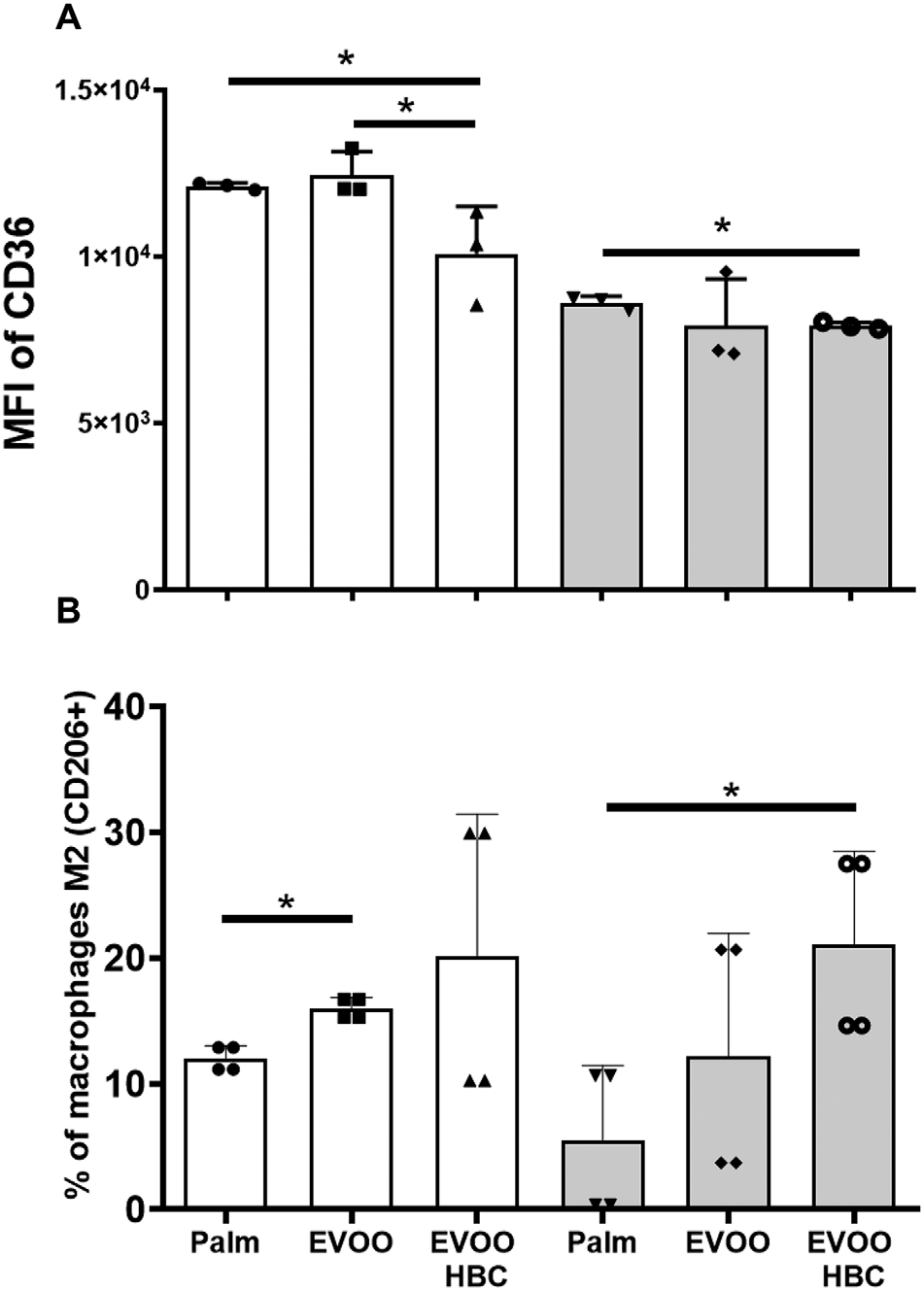
Monocyte and macrophage analysis. Panel A displays the expression of CD36 in circulating monocytes. The results are expressed as the mean fluorescence intensity of CD36 (MFI of CD36). Panel B corresponds to the percentage of peritoneal macrophages M2 (CD206+) from subsets of four determinations. The bar chart shows the mean and standard deviation for all experimental groups along the individual data. The white boxes in the graph correspond to male mice receiving palm oil, extra virgin olive oil (EVOO), and high biological compound extra virgin olive oil (EVOO HBC), while the grey boxes correspond to females. Statistical differences between groups were assessed using the Mann–Whitney *U*‐test. A *p* value less than 0.05 was considered statistically significant.

As shown in Figure 5A, the expression of CD36 as mean fluorescent intensity (MFI of CD36) in circulating monocytes was found to be lower in males fed the EVOO HBC compared to the other two experimental groups. In females, statistical significance was observed only between the palm and EVOO HBC (Figure 5A).

The percentage of peritoneal M2 macrophages was identified using CD206+ (Figure 5B). For males, the percentages of M2 macrophages were 12%, 16%, and 20.1% for palm, EVOO, and EVOO HBC, respectively. The only statistically significant difference was between palm oil and EVOO, likely due to a higher standard deviation in the EVOO HBC group. In females, the percentages of M2 macrophages were 5.5%, 12.2%, and 21%, respectively, for palm, EVOO, and EVOO HBC. There was statistical significance between palm and EVOO HBC.

### 3.6 Atherosclerotic Lesions

**FIGURE 6:**
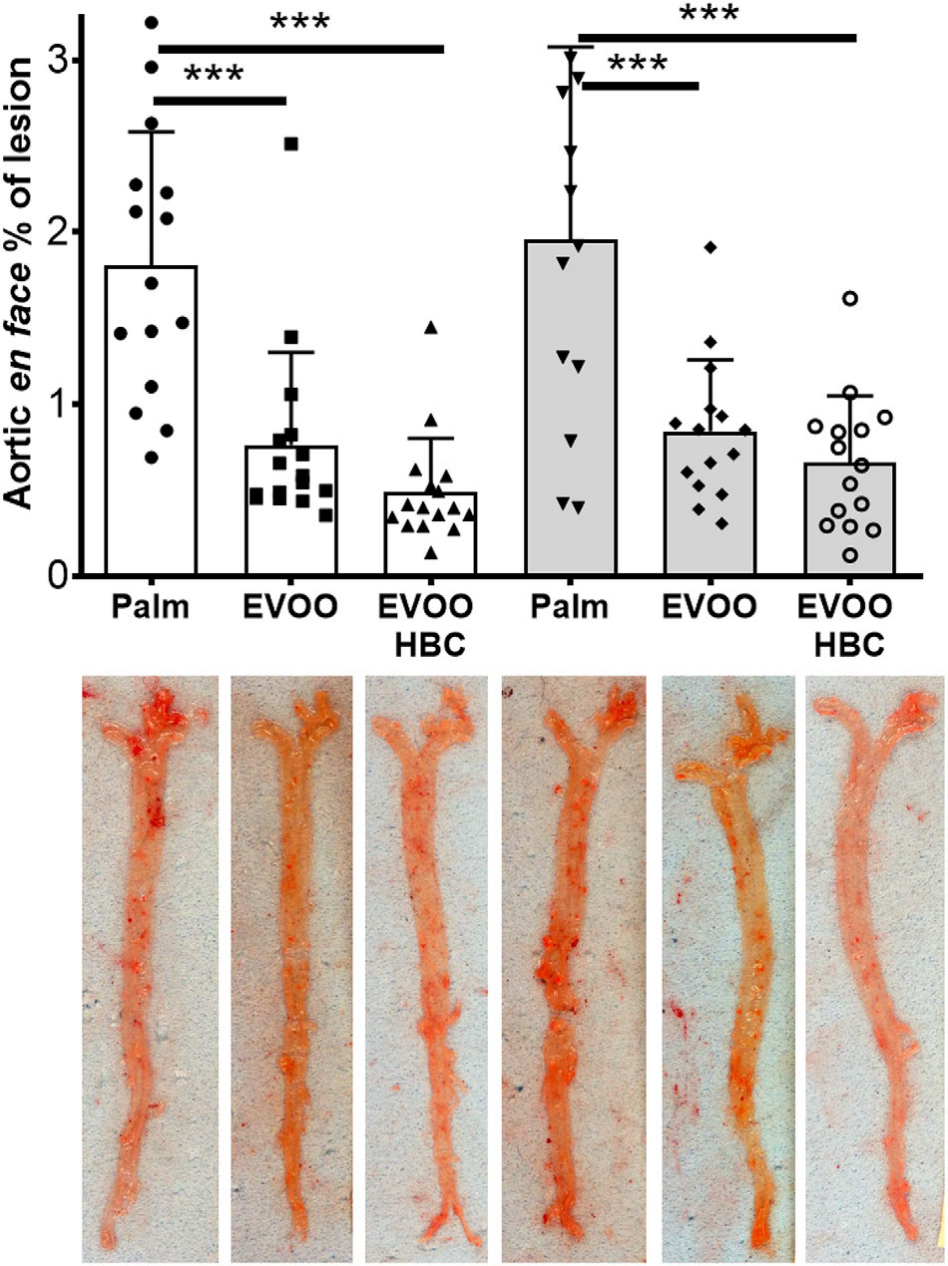
Atherosclerotic lesion of aortic *en face* lesion expressed as % of total surface. The bar chart shows the mean and standard deviation for all experimental groups along the individual data. The white boxes in the graph correspond to male mice receiving palm oil (*n* = 16), extra virgin olive oil (EVOO, *n* = 16), and high biological compound extra virgin olive oil (EVOO HBC, *n* = 16), while the grey boxes correspond to females (*n* = 15 for all groups). Statistical analysis was performed by ordinary one‐way ANOVA with Tukey multiple comparisons. The significance levels are indicated by ***, *p* ≤ 0.001.

The study showed that males and females who consumed EVOO had reduced atherosclerotic lesions in terms of the presence of foci, estimated as lipid staining of the whole aorta (Figure 6). In males, the percentage of aortic atherosclerotic area stained with lipids was found to be 1.81%, 0.76%, and 0.49% for palm, EVOO, and EVOO HBC, respectively, with statistical significance observed between all experimental groups when Mann–Whitney's *U* test was used as pair‐wise comparison or between palm and EVOO groups using one‐way ANOVA. In females, the percentages were 1.95%, 0.84%, and 0.66% for the palm, EVOO, and EVOO HBC, respectively, and statistical significance was observed only between the palm and both EVOO groups.

**FIGURE 7:**
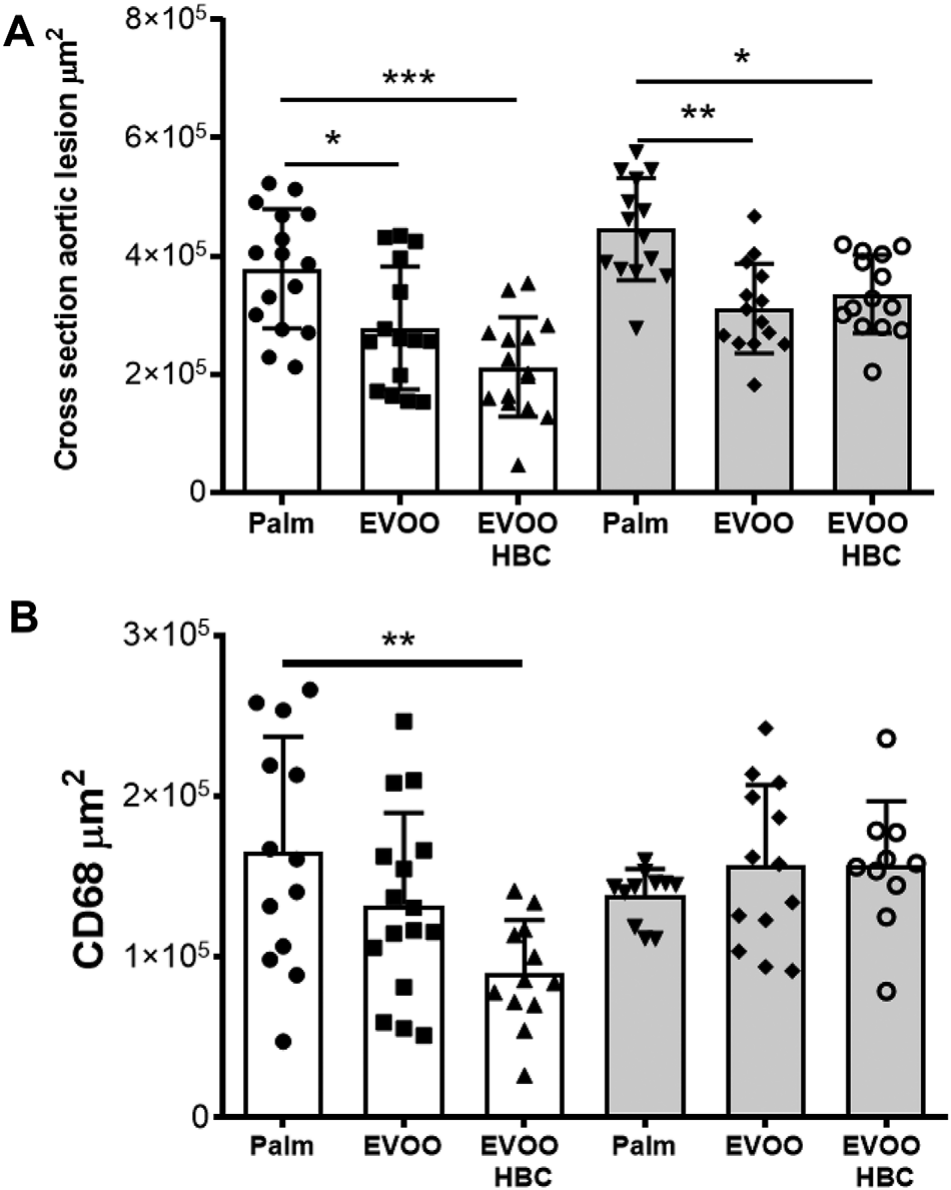
Cross‐sectional atherosclerotic aortic lesions. (A) Area occupied by lipid staining expressed as µm^2^, (B) immunohistochemistry of CD68 staining in cross‐sectional slides expressed as µm^2^. The bar chart shows the mean and standard deviation for all experimental groups along the individual data. The white boxes in the graph correspond to male mice receiving palm oil (*n* = 16), extra virgin olive oil (EVOO, *n* = 16), and high biological compound extra virgin olive oil (EVOO HBC, *n* = 16), while the grey boxes correspond to females (*n* = 15 for all groups). Statistical analysis was performed by ordinary one‐way ANOVA with Tukey multiple comparisons. The significance levels are indicated by * for *p* ≤ 0.05, ** for *p* ≤ 0.01, and *** for *p* ≤ 0.001.

In males, plaque growth, estimated by the cross‐sectional area of the aortic root (Figure 7A), was smaller and statistically significant in both extra virgin olive oil (EVOO) groups compared with the palm group. This finding is consistent with the *en face* procedure. In females, similar findings were observed. Presence of macrophages in the atherosclerotic plaques was evaluated by CD68 staining (Figure 7B). The areas occupied by these cells were significantly elevated in males from the palm group compared to the EVOO HBC group. No such pattern was observed in females.

**FIGURE 8:**
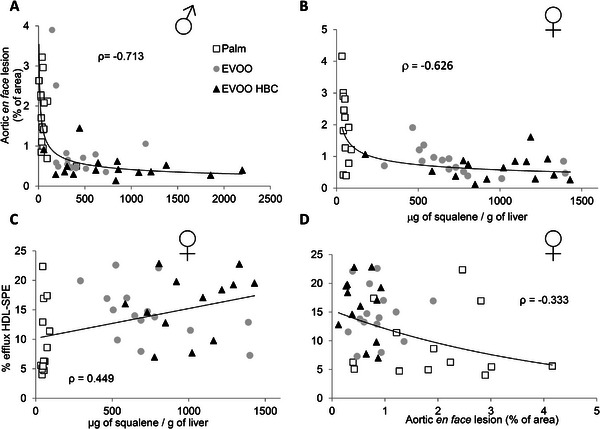
Relationship between the *en face* percentage of lesions in aortas and the amount of squalene per gram of liver tissue in males (A) and in females (B). The association between the percentage of efflux HDL‐SPE and the amount of squalene per gram of liver tissue in females (C), and the percentage of efflux HDL‐SPE and the percentage of lesions in their aortas in females (D). The obtained Spearman's *ρ* values, with a *p* value <0.01, are shown.

Association among different parameters through significant correlations is displayed in Figure 8. An inverse correlation was observed between *en face* aortic lesions and squalene liver content in both sexes (Figure 8A,B) with Spearman's *ρ* values of −0.713 and −0.626, respectively. Furthermore, in females, the percentage of efflux HDL‐SPE exhibited a positive correlation with the amount of liver squalene content and a negative with en face aortic lesions with Spearman's *ρ* values of 0.449 and −0.333, respectively (Figure 8C,D).

## 4 Discussion

**FIGURE 9:**
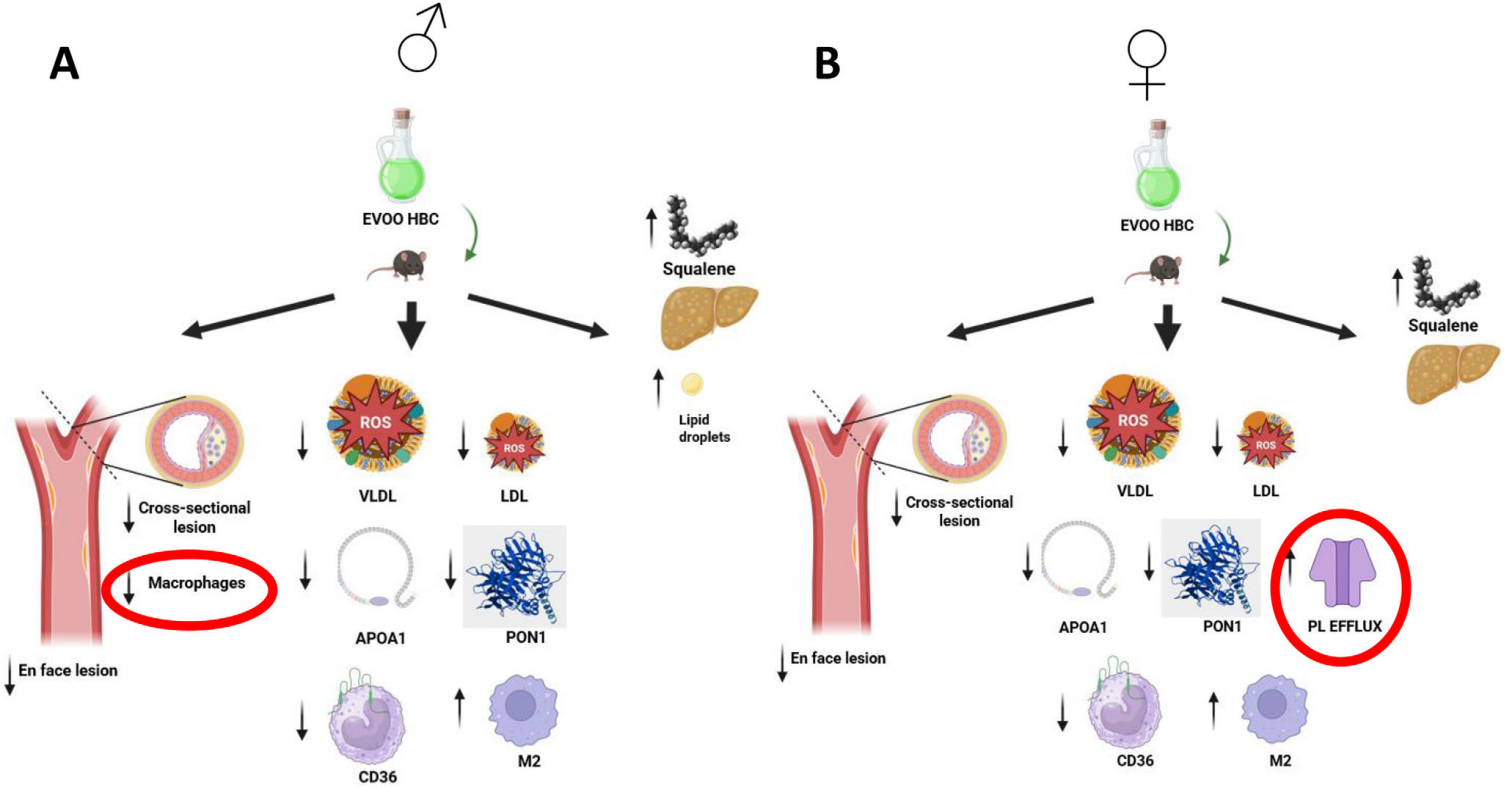
Comprehensive scheme displaying the observed findings. A, males and B, females. Created in Biorender.com (accessed on May 1, 2025).

The present study was conducted to investigate the relevance of a particular enrichment in squalene and the dialdehyde form of decarboxymethyl oleuropein aglycone in EVOO on the atherosclerosis development in *Apoe*‐deficient mice. Somatometric parameters indicated that these EVOOs were well tolerated. Accordingly, the higher supply of squalene was translated into its accumulation in the liver. No impact on plasma cholesterol but increases in TG and glucose, and decreases in APOA1 and paraoxonase 1 were observed in the groups receiving both EVOO. Despite these findings, the oxidative status of lipoproteins and HDL‐phospholipid efflux were more favorable in the groups consuming EVOO. The expression of CD36 in circulating monocytes was also decreased by this new EVOO HBC. Likewise, the presence of M2 in peritoneal macrophages was enhanced by this EVOO HBC. Concomitantly, the presence of atherosclerotic foci was reduced by the inclusion of these new components. Furthermore, the presence of plaque macrophages was reduced by this new oil, but only in males. HDL‐phospholipid efflux was negatively correlated with the presence of atherosclerotic *foci*, but only in females. These findings indicate that the inclusion of these compounds in EVOO ameliorates the atherosclerotic effect through plaque macrophage involvement or HDL functionality, depending on sex (Figure 9).

The content of squalene in EVOO HBC was 13 620 mg/kg. This represents a high value not commonly observed in EVOO [31]. The second main enriched component was the dialdehyde form of decarboxymethyl oleuropein aglycone (3, 4‐DEPEH‐EDA), 30 times higher in EVOO HBC than EVOO (Table 1). The fact that these changes were obtained by a new mechanical procedure that favors their enrichment allows the use of EVOO according to the current European law [32]. Although not distinctive between both EVOOs, the contents of oleocanthal and oleacein were also elevated compared to those reported (0.2–498 mg/kg) [33]. Likewise, alpha‐ and gamma‐tocopherols were clearly higher than their currently reported values (10.2–320 and 0.7–8.8 mg/kg, respectively) [34]. These findings are particularly pertinent in light of the European Commission's approval in 2012 of the statement “olive oil phenols contribute to the protection of blood lipids from oxidative stress” for use on extra virgin olive oil [32]. However, other phenolic compounds were lost in EVOO HBC, including lignans, oleuropein aglycone, ligstroside aglycone, hydroxytyrosol, tyrosol, cyanidin chloride, ferulic acid, and vanillic acid. This special mixture enables the in vivo relevance of the described enrichment to be tested, which may compensate for the loss, according to the results.

No single mechanism seems to explain all the changes induced by squalene, although it has been proposed that squalene acts as a peroxisome proliferator‐activated receptor α (PPARα) agonist [35]. The extent and potency of this agonist have not yet been tested. Furthermore, considering that PPARα dimerizes with other transcription factors and interacts with numerous coactivator proteins [36], the field is open to complex mechanisms that explain the wide range of observed effects [37, 38]. The dialdehyde form of decarboxymethyl oleuropein aglycone (3,4‐DEPEH‐EDA) was evaluated in vitro as a molecule that could be absorbed, with the potential to exert anti‐inflammatory effects by inhibiting cyclooxygenase 1 and 2 [39]. Further research is warranted on the synergy of both compounds.

As expected, the higher supply of squalene in the diet was translated into a higher hepatic content of this compound (Figure 1). When the area of lipid droplets was quantified, an increase was also observed (Figure 2). This finding is in agreement with previous results in the sense that hepatic lipid droplets accumulate squalene when mice were fed diets enriched with pure squalene [37, 38] and this is an effect depending on animal model [28]. The hepatic increase in lipid droplets was also observed in these mice when consumed EVOO of different cultivars when compared to palm oil [40]. Interestingly, the hepatic squalene contents observed in mice consuming the EVOO HBC were 10 times higher than those reached when received 1 g/kg of squalene added to Western diets [37]. However, the dose of squalene provided was only of 39 mg/kg, 50 times lower, which indicates that this matrix of EVOO was highly efficient to favor the intake of squalene. Considering the higher metabolic rate of mice [41], the latter dose would translate into a human of 3.9 mg/kg/day. This is slightly lower than the used in human nutritional studies (15 mg/kg/day) [42]. Thus, the present study uses a dose comparable to those provided in human nutritional interventions. This fact poses the following two important consequences: first, using this nutraceutical approach, a pharmacological dose is reached, and second, humans consuming these oils, if they accumulate squalene in their livers, they could be misclassified as displaying metabolic associated steatotic liver disease (MASLD). According to recent findings, the cholesterol content of lipid droplets seems crucial to liver damage [43]. In this sense, the accumulation of squalene in lipid droplets could disrupt the incorporation of cholesterol. In vivo and in vitro evidences point out that hepatic squalene accumulation may play a beneficial role in reducing endoplasmic reticulum and oxidative stresses [44, 45, 46] and sharks that do accumulate in the liver live for centuries [47].

No impact was observed on total plasma cholesterol (Table 1), what could be justified by the different lipoprotein profiles (Figure S2). The palm group increased VLDL/rQm and decreased LDL, while the opposite occurred in the EVOO groups. These groups also showed increased plasma TG, something also observed in pigs receiving squalene [48], but not in *Apoe*‐deficient mice consuming different EVOO [49]. The increase in TG represents a redistribution of these compounds and cholesterol in the different lipoproteins although of lesser magnitude than the observed in rabbits consuming squalene [50]. There was an increase in glucose in the EVOO groups (Table 2). Decreases in APOA1 and paraoxonase 1 were also observed in the groups receiving both EVOO (Figure 4A,B). A similar trend was observed in *Apoe*‐deficient mice receiving Arbequina cultivar EVOO compared to palm oil [49] and in these female mice receiving squalene [27]. Despite these findings, the oxidative status of lipoproteins was more favorable in the groups consuming EVOO (Figure 3). Oxidized low‐density lipoproteins through endothelial cells LINC00657 expression, a long noncoding RNA, favored angiogenesis that promoted plaque growth, causes plaque hemorrhage, and plaque instability [51]. A decrease in oxidative stress in *Apoe*‐deficient mice consuming squalene and the absence of changes in total paraoxonase activity were previously observed [44]. Three factors may be involved, on one hand, the cargo of antioxidants in these lipoproteins. In this regard, Ruiz‐Garcia et al. observed that the consumption of EVOO enriched in oleocanthal and oleacein by obese and diabetic patients improved their oxidative status [52]. Notably, the EVOO employed in this study contained higher concentrations of both compounds (Table 1). Another aspect to be considered is the fact that paraoxonase changes its lipoprotein distribution in function of diets [49], an aspect not addressed in the present research. Third, the open debate regarding to which extent plasma APOA1 and HDL‐cholesterol levels represent the HDL functionality [26]. These authors have proposed a simplified method to explore cholesterol efflux using a cell‐free assay. In this report, this method has been adapted to mice, and HDL‐phospholipid efflux was found to be more favorable in the female groups consuming EVOO (Figure 4C). A consumption of phenol‐enriched virgin olive oil in female C57BL/6 mice also increased the reverse cholesterol transport in vivo [53], an aspect not addressed in the present report. These observations point out to a sex‐specific difference in the reverse cholesterol transport using EVOO and add further complexity to the differences in HDL levels between sexes. Since the Zhao et al. assay is cell‐free, some of the protein and lipid [54] components of HDL may be involved. APOA1 changes (Figure 4A) do not explain the changes since its levels decreased in both sexes. Potential candidates to be explored are numerous, considering that HDL carries more than 110 proteins [55] and whose sex and diet regulation awaits further research. These plasma findings indicate that plasma lipids are quite sensitive to the composition of EVOO oils, to the animal model and the experimental setting, and there was a trade‐off of opposite plasma findings.

Atherosclerosis is a complex entity where not only plasma components and functionality are involved but also inflammatory cells play an active role, particularly monocytes/macrophages [56]. The activation of circulating monocytes, estimated by the expression of CD36, was found to be significantly decreased by consumption of EVOO HBC and in males between both types of EVOO (Figure 5A). The decrease in this scavenger receptor could be associated with the observed decrease of oxidized lipoproteins (Figure 3A) since these particles elicit CD36 expression [57]. Interestingly, Granados‐Principal et al. found that squalene in vitro reduced CD36 scavenger receptor expression in macrophages [58]. The use of CD36 MFI as a sole marker of monocyte activation may be insufficient and future work using other markers (e.g., CD14, CD16) should be considered. Macrophages can differentiate into proinflammatory M1 or anti‐inflammatory M2 phenotypes, and this process is pivotal for immune system function, tissue defense, and damage repair [59]. M2 subtypes suppress inflammation in atherosclerotic plaques and favor plaque stability [60, 61] or protect viral myocarditis [62]. In this dietary intervention, the percentage of macrophages M2 (Figure 5B) increased in males consuming EVOO compared to palm diet, and in females the effect of EVOO HBC was even more pronounced that of EVOO. These findings indicate that activation of circulating monocytes and polarization into the M2 phenotype are influenced by the presence of biological components of EVOO, and the outcome is sex‐dependent. A limitation of these macrophage analyses is that they are based on small pooled samples and should be expanded in future studies.

In the present report, the presence of atherosclerotic *foci* and the growth of plaques in the aorta were quantified using *en face* and cross‐sectional lesion analyses, respectively (Figures 6 and 7A). It is noteworthy that only the *en face* method exhibited statistically significant differences between the male mice consuming EVOO and EVOO HBC. This indicate that the presence of *foci* was reduced by the presence of squalene and the dialdehyde form of decarboxymethyl oleuropein aglycone in EVOO. In this sense, Bullon et al. observed a reversal of vascular damage in rabbits receiving squalene [63]. When the growth of atherosclerotic plaques was analyzed at the aortic roots using the cross‐sectional procedure, a reduction in the EVOO‐consuming groups was observed in both sexes (Figure 7A). This finding reinforces the described antiatherosclerotic properties of EVOO when compared with palm oil [49] and extend the previous result to both sexes. Furthermore, the presence of plaque macrophages, assayed as CD68 immunohistochemistry (Figure 7B), was significantly reduced only in males consuming EVOO HBC. This is in contrast with the decrease observed in females when using MOMA staining [49]. This would be in agreement with the proposed relationship between CD68 and oxidized LDL [64], but only in males, and could influence plaque stability [65]. Other compounds, such as astragaloside IV, inhibit macrophage proliferation and migration [66]. These issues raise the question of whether there are different populations of plaque macrophages depending on sex [67] and whether these populations are modulated by the consumption of EVOO.

In conclusion, the elevated content of squalene and the dialdehyde form of decarboxymethyl oleuropein aglycone in EVOO HBC consumed by *Apoe*‐deficient mice was translated into a reduction of atherosclerotic *foci* and growth of existing plaques in both sexes (Figure 9). These findings were accompanied by the absence of changes in plasma total cholesterol, increases in TG and glucose, decreases in APOA1 and paraoxonase 1, reduced lipoprotein oxidation, decreased expression of CD36 in circulating monocytes, increased differentiation of peritoneal macrophages into M2 phenotype, and hepatic accumulation of squalene. A differential sex involvement was observed regarding a reduced presence of CD68‐expressing macrophages in males and a reduction of atherosclerotic *foci* negatively correlated with HDL‐phospholipid efflux in females. Thus, the enrichment of EVOO in these components modulates the recruitment of macrophages or improves HDL efflux in a sex‐dependent way.

## Conflicts of Interest

The company Cleanthi Alpha Olenic LTD (Larnaca, Cyprus) provided the extra virgin olive oils utilized in the experimental research and their analyses. No further funding was received from the company, and this has no involvement in the analysis of results and the decision of publish.

## Declaration of Generative AI and AI‐Assisted Technologies in the Writing Process

During the preparation of this work, the authors used DeepL Write (DeepL SE, Cologne, Germany) in order to improve English use. After using this tool, the authors reviewed and edited the content as needed and takefull responsibility for the content of the publication.

## Supporting information




**Supporting File 1**: mnfr70223‐sup‐0001‐SuppMat.docx

## Data Availability

Data will made available to scientists on reasonable request.
