# Supplementary material for: Effect of Extra Virgin Olive Oil High in Bioactive Compounds on Atherosclerosis in Apoe‐Deficient Mice
Source: Mol Nutr Food Res. 2025 Sep 1;69(22):e70223. doi: 10.1002/mnfr.70223 (PMC12643192; doi:10.1002/mnfr.70223)
Supplement: Supplementary file 1 — Supporting File 1: mnfr70223‐sup‐0001‐SuppMat.docx [file MNFR-69-e70223-s001.docx]

Figure S1. Feed intake of males (A) and females (B), body weight gain (C) and liver weight (D). White boxes correspond to males and grey boxes correspond to females. Ordinary one-way ANOVA with Tukey’s multiple comparisons and statistical significance with * P<0.05.

**B**

**A**

**D**

**C**

Figure S2: Effects of different diets on plasma lipoproteins in *Apoe*-deficient mice. Plasma lipoproteins were separated by FPLC and the collected fractions were analyzed for total cholesterol in A for males and B for females.

*****

**A**

HDL

VLDL/rQm

LDL

**B**
